# Molecular Characterization and Expression Analysis of a Gene Encoding 3-Hydroxy-3-Methylglutaryl-CoA Reductase (HMGR) from *Bipolaris eleusines*, an Ophiobolin A-Producing Fungus

**DOI:** 10.3390/jof10070445

**Published:** 2024-06-26

**Authors:** Jianping Zhang, Ke Yang, Wei Tang, Yongjie Yang, Xiaoyue Yu, Yongliang Lu, Liuqing Yu

**Affiliations:** 1 State Key Laboratory of Rice Biology and Breeding, China National Rice Research Institute, Hangzhou 310006, China; 2 Department of Industrial Engineering, University of Arkansas, Fayetteville, AR 72701, USA; ky013@uark.edu

**Keywords:** cloning, RACE technology, transcriptome sequencing, methyl jasmonate (MJ), phytotoxin

## Abstract

Ophibolin A, a fungal sesterterpene, exerts a pivotal influence in a diverse array of biological processes, encompassing herbicidal, bactericidal, fungicidal, and cytotoxic activities. Sixty genes associated with sesterterpene compound biosynthesis were obtained from *Bipolaris eleusines* via transcriptome sequencing, and those closely linked to ophiobolin A biosynthesis were subsequently filtered. A gene encoding 3-hydroxy-3-methylglutaryl-CoA reductase (HMGR) that catalyzes the first committed step of ophiobolin biosynthesis in the mevalonic acid (MVA) pathway was isolated and characterized using RACE (Rapid Amplification of cDNA Ends) technology from ophiobolin A-producing fungus, *B. eleusines*. The full-length cDNA of the *B. eleusines HMGR* gene (*BeHMGR*) was 3906 bp and contained a 3474 bp open reading frame (ORF) encoding 1157 amino acids. Sequence analysis revealed that deduced BeHMGR had high homology to the known HMGRs from *Pyrenophora tritici-repentis* and *Leptosphaeria maculans*. It had a calculated molecular mass of about 124.65 kDa and an isoelectric point (pI) of 6.90. It contained two putative HMG-CoA-binding motifs and two NADP(H)-binding motifs. Induced expression analysis of the *BeHMGR* gene by methyl jasmonate treatment using quantitative fluorescence PCR showed that it significantly elevated after 3 h of methyl jasmonate treatment, peaked at 6 h, and then gradually decreased. This demonstrates that *BeHMGR* gene expression is induced by methyl jasmonate.

## 1. Introduction

*Bipolaris eleusines*, obtained from naturally infected barnyard grass (*Echinochloa crus-galli*), was evaluated as a potential biological control agent for barnyard grass [1]. It produces ophiobolin A, a sesquiterpenoid with a tricyclic structure known for its herbicidal [2,3], antimicrobial [4,5], and anti-tumor cell activities [6,7,8,9], holding promising applications in agriculture and medicine. Previous studies have focused on isolating ophiobolin toxins and investigating their functions [2,3,4,5,10,11,12].

Ophiobolin A, a secondary metabolite produced by plant pathogenic fungi, is synthesized in small quantities, limiting its commercial production. Chemical synthesis could address the challenge of low microbial secondary metabolite production, but its complex compound structure and high synthesis costs pose limitations to its industrial-scale production [13,14,15]. Alternatively, toxin production can be enhanced through methods such as mutagenesis. Our laboratory has employed various techniques, including chemical mutagenesis, ultraviolet mutagenesis, protoplast fusion technology, and Restriction enzyme-mediated integration (REMI) technology, to increase toxin production [16,17]. Despite screening some mutant and fusion strains with improved toxin production, they have not yet met the requirements for industrial-scale production.

Gene regulation might enhance toxin production in *B. eleusines*. As a significant terpenoid, the biosynthesis pathway of ophiobolin A closely resembles that of other terpenes in fungi, operating via the mevalonate pathway [18,19]. This pathway initiates from acetyl-CoA and proceeds through a series of enzymatic reactions to produce 3-hydroxy-3-methylglutaryl coenzyme A (HMG-CoA), farnesyl pyrophosphate (FPP), geranylgeranyl pyrophosphate (GGPP), and geranyl farnesyl pyrophosphate (GFPP). These intermediates then undergo cyclization and a sequence of redox reactions to form mature terpenoid structures.

Among these, 3-hydroxy-3-methylglutaryl-CoA reductase (HMGR) catalyzes the NADPH-dependent conversion of HMG-CoA to mevalonate (MVA) [20]. As the synthesis of mevalonate is an irreversible process, HMGR is recognized as the primary rate-limiting enzyme in the mevalonate biosynthetic pathway across animals, plants, and fungi [21,22,23]. It also serves as a crucial target enzyme in intracellular terpenoid metabolism pathways. Due to HMGR’s pivotal role in the biosynthesis of significant terpenoids, such as paclitaxel, artemisinin, tanshinone, and ganoderic acid, this gene has been extensively cloned and characterized from many organisms [24,25,26,27]. However, no reports have concerned an ophiobolin A-producing fungus, *B. eleusines*. Hence, this study utilized Rapid Amplification of cDNA Ends (RACE ) technology to clone the *HMGR* gene (referred to as *BeHMGR*) from the ophiobolin A-producing plant pathogenic fungus, *B. eleusines*. Additionally, gene expression was analyzed using fluorescence quantitative PCR after methyl jasmonate treatment. The objective of this work is to provide a pivotal enzyme target for further investigation into the molecular mechanisms and metabolic regulation of the ophiobolin A biosynthetic pathway, thereby laying the groundwork for future studies on large-scale toxin synthesis through metabolic regulation.

## 2. Materials and Methods

### 2.1. Fungal Inoculum

*Bipolaris eleusines* (Alcorn & Shivas) was isolated from severely diseased barnyard grass and stored in PDA (potato 20%, glucose 2%, and 1.5% agar powder) at 4 °C in the Weed Laboratory at the China National Rice Research Institute, Hangzhou, China [1]. *Escherichia coli*, JM109 was purchased from Sangon Biotech (Shanghai) Co., Ltd., Shanghai, China.

A fungal-activated inoculum was transferred from the vigorous edge growth of *B. eleusines* into the center of a fresh soybean medium (glucose 3%, soybean flour 4%, MgSO_4_ 0.1%, Na_3_PO_4_·12H_2_O 0.2%, KNO_3_ 0.5%, and agar powder 1.8%) plate (φ9 cm) using a fungal block (φ5.5 mm). The inoculum was incubated in the dark at 28 °C for 4 days to extract total RNA for subsequent steps.

### 2.2. Transcriptome Sequencing

RNA preparation, transcriptome sequencing, and De Novo analysis were conducted at the *BGI Genomics* Co., Ltd., Shenzhen, in China using Illumina HiSeq™ 2000.

### 2.3. Cloning of the Full-Length cDNA of BeHMGR by RACE

#### 2.3.1. Confirmation of EST Amplification from *B. eleusines*

Total RNA was extracted from *B. eluesines* using the PureLink^TM^ RNA Mini Kit following the manufacturer’s instructions (Invitrogen, Carlsbad, CA, USA). Single-stranded cDNAs were synthesized from 5 μg of total RNA with an oligo (dT)17 primer according to the manufacturer’s protocol (SuperScript^®^ II Reverse Transcriptase, Invitrogen, Carlsbad, CA, USA). Following S.N.A.P.™ column treatment, the single-stranded cDNA mixture served as the template for PCR to confirm the amplification of the EST from *B. eleusines.* This verification employed the primers *BeHMGR*-EST-F and *BeHMGR*-EST-R (Table 1) designed based on the EST sequences obtained from transcriptome sequencing.

#### 2.3.2. 5′ End Amplification of *BeHMGR* Gene

The 5′ RACE method was utilized to amplify the 5′ end of the *BeHMGR* gene. Initially, the SuperScript^®^ II Reverse Transcriptase (Invitrogen™) enzyme and primer *BeHMGR*5GSP-1 (Table 1) were employed to synthesize the first-strand cDNA of the target gene from total RNA. Following treatment with RNase Mix and subsequent purification using the DNA Purification System (GLASSMAX DNA isolation spin cartridges), PolyC residues were added to the ends of the purified single-stranded cDNA using the TdT enzyme and dCTP. In conjunction with the specific primer *BeHMGR*5GSP-2 (Table 1), the bridging primer AAP provided in the kit facilitated the first round of PCR amplification of the cDNA extended with a dC tail. For the second round of nested PCR amplification, the bridging amplification primer AUAP included in the kit and specific primer *BeHMGR*5GSP-3 (Table 1) were utilized. The PCR products from the second round were subjected to electrophoresis, and the target band was isolated and purified from the gel. Subsequently, the purified PCR product was sub-cloned into the pMD18-T vector, and positive clones were selected for sequencing. 

#### 2.3.3. 3′ End Amplification of *BeHMGR* Gene 

The reverse transcriptase SMARTScribe™ Reverse Transcriptase and primer 3′ CDS primer A were employed to reverse-transcribe total RNA, synthesizing cDNA. For the first round of PCR amplification, the specific primer *BeHMGR*3-1 (Table 1) and universal UPM were utilized, with the synthesized cDNA as the template. Subsequently, the products from the first round of PCR amplification were diluted 50 times, and the second round of PCR amplification was carried out using the primer *BeHMGR*3-2 (Table 1) and universal UPM. The purified PCR product from the second round was sub-cloned into the pMD18-T vector for sequencing.

#### 2.3.4. Obtaining Full-Length cDNA of *BeHMGR* Gene

The amplified 3′ and 5′ RACE cDNA sequences, along with the EST sequences, were assembled using Vector NTI Advance 10 software to generate the full-length cDNA sequence of the target gene. Subsequently, based on this assembled sequence, a pair of gene-specific primers, *BeHMGR*-F and *BeHMGR*-R (Table 1), were designed to amplify the full-length cDNA. The PCR product was confirmed by sequencing. The sequence obtained through sequencing was submitted to the National Center for Biotechnology Information (NCBI), and blast analysis was conducted on its open reading frame (ORF), start codon, stop codon, and other information. Once the analysis was completed, the sequence was then submitted to the NCBI (GenBank accession No. JQ780844).

### 2.4. Bioinformatics Analysis 

Bioinformatic analysis of *BeHMGR* was conducted online using the websites http://www.ncbi.nlm.nih.gov (accessed on 1 February 2024) and http://cn.expasy.org (accessed on 3 February 2024). Sequence alignment and assembly were performed using Vector NTI Advance 10 software, while multiple sequence alignment was carried out using DNAMAN 8.0 software. ORF, GenBank BLAST, and protein sequence searches were executed on the NCBI website [http://www.ncbi.nlm.nih.gov (accessed on 1 February 2024)]. ORF translation was completed using DNAStar Lasergene 14.1 software. 

### 2.5. Expression Analysis of BeHMGR Gene under Methyl Jasmonate Treatment

#### 2.5.1. Methyl Jasmonate Treatment Procedure for *B. eleusines*

A fungal block (φ5.5 mm) was used to transfer an inoculum from the vigorous edge growth of *B. eleusines* into a 250 mL Erlenmeyer flask containing 100 mL of PDB medium. The inoculum was cultured on a 120 rpm shaker at 28 °C for 7 days. Then, a 5% inoculation volume of *B. eleusines* was taken from the above culture and inoculated into 50 mL of PDB liquid medium, where it was cultured for 60 h. Methyl jasmonate (a final concentration of 2 mM) was added to the fungal culture fluid as induction treatment, and culturing was continued. Mycelia were collected at intervals of 0.25 days, 1 day, 2 days, 3 days, 4 days, and 5 days for the analysis of the *BeHMGR* gene expression level.

#### 2.5.2. Fluorescent Quantitative PCR Detection of *BeHMGR* Gene Expression Level

Total RNA was extracted from *B. eleusines* strains treated with methyl jasmonate at different time intervals and reverse-transcribed into first-strand cDNA. The expression levels of the *BeHMGR* gene were detected using fluorescent quantitative PCR. The primer sequences used can be found in Table 1. The real-time PCR amplification protocol was as follows: Stage 1: Pre-denaturation at 95 °C for 2 min, followed by 95 °C for 10 min. Stage 2: PCR reaction, consisting of 40 cycles of 95 °C for 15 s, 60 °C for 40 s, and 72 °C for 40 s. Stage 3: Dissociation curve analysis, consisting of 95 °C for 15 s, 60 °C for 1 min, 95 °C for 15 s, and 60 °C for 1 min. Three biological replicates were set for each treatment, and each sample was analyzed thrice.

#### 2.5.3. Data Analysis and Statistics

The real-time quantitative PCR data were analyzed using the 2^−ΔΔct^ method. Statistical analysis was performed using an SPSS 13.0 statistical package. The statistical data, presented as mean ± standard deviation (SD), were analyzed for the significance of difference (*p* < 0.05) using a standard variance analysis with a completely randomized experimental design, followed by Tukey’s multiple-range tests at a 5% significance level.

## 3. Results and Analysis

### 3.1. Results of Transcriptome Sequence Analysis of B. eleusines

Transcriptome sequencing yielded a total of 26,555,560 high-quality ESTs (i.e., total reads) with a total nucleotide count of 2,390,000,400 and a Q20 score of 92.91%, indicating a very high sequencing quality (Table 2). After clustering and assembly, 32,100 high-quality consensus sequences (Unigenes) were obtained, and the total nucleotide count of the consensus sequences reached 17,959,906 (Table 3). Among these, there were 20,075 consensus sequences in the range of 100-500 nt, representing 62.54% of the total, and 466 consensus sequences greater than 2000 nt, accounting for 1.45% of the total. The N50 of the consensus sequences is 743 nt, and the average length is 559 nt.

**Figure 1:**
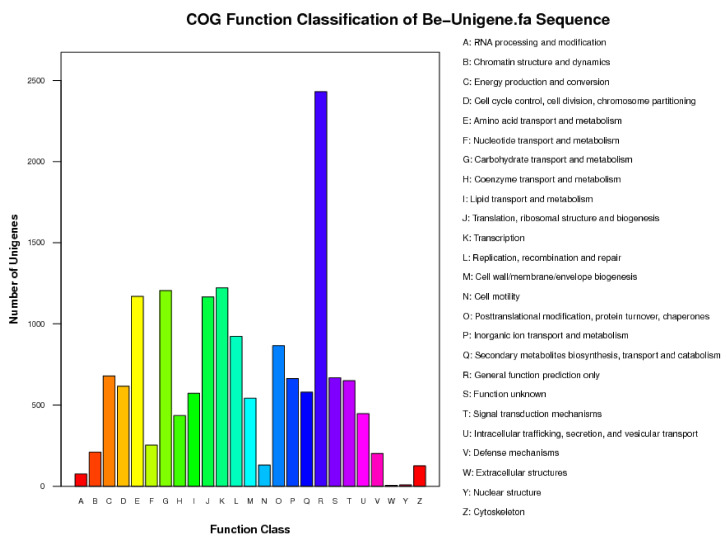
Data of COG classification.

The consensus sequences obtained from clustering and assembly with the COG database were compared to predict the possible functions of the consensus sequences and perform functional classification statistics on them. A total of 31,432 sequences had homologous sequences, with an annotation rate of 97.9%. The remaining 668 sequences were of an unknown function, from which new genes may be discovered. The annotated genes were functionally classified into 26 categories (Figure 1). The largest number of sequences were mainly concentrated in protein sequences with only general function predictions (2341), transcription-related sequences (1221), amino acid transport and metabolic sequences (1169), and sequences involved in translation, ribosome structure, and biogenesis (1165). Additionally, there were 579 sequences consistent with the categories we focused on for secondary metabolite biosynthesis, transport, and catalysis, as well as some sequences related to protein modification, folding, and molecular chaperones (865), coenzyme transport and metabolism (435 sequences), and sequences related to signal transduction mechanisms (650 sequences), which also attracted our attention.

KEGG Pathway analysis identified 60 genes in the terpenoid backbone biosynthesis pathway within the transcriptome sequence, which are the gene sequences essential for our research. The genes in this pathway include Farnesyl pyrophosphate synthase (*FPPS*), Acetyl-CoA acetyltransferase (*AAT*), Hexaprenyl pyrophosphate synthase (*HPS*), Isopentenyl-diphosphate delta-isomerase (*IDDI*), Geranylgeranyl pyrophosphate synthase (*GGPPS*), 3-hydroxy-3-methylglutaryl CoA synthase (*HMGS*), 3-hydroxy-3-methylglutaryl CoA reductase (*HMGR*), Diphosphomevalonate decarboxylase (*DMD*), Mevalonate kinase (*MK*), Phosphomevalonate kinase (*PMK*), 3-ketoacyl-CoA thiolase B (*KT*), and 17 other genes.

Additionally, 161 other genes related to terpenoid metabolism pathways were found, including 90 genes involved in the sterol biosynthesis pathway and genes related to quinone and another terpenoid–quinone biosynthesis. Sixty-four genes were found, including seven genes in the carotenoid biosynthesis pathway. All these compounds share isoprene as their basic unit. Moreover, 76 transcription factor-related sequences were discovered.

### 3.2. Molecular Characterization of HMGR Gene of B. eleusines

3′RACE and 5′RACE were employed to clone the 3′ end and 5′ end of the *HMGR* gene. Primers were designed based on the EST sequence of the *HMGR* gene in the *B. eleusines* transcriptome, and 5′RACE and 3′RACE kits were used to amplify the 5′ and 3′ ends of *HMGR*, respectively. The PCR product bands obtained from the second round of PCR amplification of 5′RACE and 3′RACE are greater than 2000 bp and about 500 bp, respectively. The target bands from electrophoresis were excised from the gel, recovered, purified, and sequenced. The BLAST results of the obtained sequences in the GenBank database show high homology with the fungal *HMGR* gene, confirming that they are the correct 3′ and 5′ ends of the *BeHMGR* gene.

The EST sequence, the 3′ end, and the 5′ end of the *BeHMGR* gene amplified by RACE were assembled to obtain the deduced full-length cDNA sequence, which was then confirmed by sequencing, along with its open reading frame, start codon, and stop codon. The analysis reveals that the full-length *BeHMGR* cDNA sequence (GenBank accession No. JQ780844) is 3906 bp long and contains an ORF of 3474 bp, which translates into a polypeptide chain of 1157 amino acids (Supplementary File S1), similar in length to polypeptide chains in other fungi. The full-length *BeHMGR* cDNA includes a 189 bp 5′-untranslated region (UTR, from 1 bp to 189 bp) and a 223 bp 3′-untranslated region downstream of the ORF (3664 bp to 3886 bp), followed by a 20 bp poly(dA) tail.

### 3.3. Bioinformatics Analysis of BeHMGR Protein

#### 3.3.1. Analysis of Physical and Chemical Properties of BeHMGR Protein

The Computer pI/Mw Tool [https://web.expasy.org/compute_pi/ (accessed on 12 February 2024)] was used to analyze and estimate that the molecular weight (MW) and theoretical isoelectric point (pI) of the BeHMGR protein are 124.65 kDa and 6.90, respectively. The half-life of the protein is 10–30 h, and the instability index is 43.12, indicating that it is an unstable protein. The aliphatic index is 92.59, and the Grand Average of Hydropathy (GRAVY) is 0.066. The number of negatively charged amino acid residues (Asp + Glu) is 105, and the number of positively charged amino acid residues (Arg + Lys) is 103.

#### 3.3.2. Conservation Analysis of BeHMGR Protein Sequence

The isolated and amplified full-length cDNA sequence of *BeHMGR* and its deduced amino acid sequence were deposited in the GenBank database. The protein–protein BLAST analysis results show that the deduced amino acid sequence of BeHMGR shares extensive and varying degrees of similarity with HMGRs from other fungi. It has 93% similarity and 86% identity with *Pyrenophora tritici-repentis* XP001941036, 90% similarity and 82% identity with *Leptosphaeria maculans* CBX91449, and 86% similarity and 78% identity with *Phaeosphaeria nodorum* XP001800116. The multiple sequence alignment analysis of BeHMGR and HMGR amino acid sequences from ten other fungi is shown in Supplementary File S2.

By analyzing multiple alignments of the amino acid sequences of BeHMGR and ten other fungal HMGRs, it was found that the N-terminal composition and length of the BeHMGR protein vary significantly, while the functions of some amino acids at the C-terminal are highly conserved, affecting the conformation and catalytic properties of the protein. 

The highly conserved regions of the C-terminal amino acids include two NADPH-binding motifs, DAMGMNM and GTIGGGT, and two HMG-CoA-binding motifs, ENV(V/I)GY(L/M)PLP and TTEGVLVA (see Supplementary File S2). Among them, glutamic acid in TTEGVLVA plays a crucial role in HMGR catalysis. The NADPH-binding domain and HMG-CoA-binding domain of BeHMGR share almost identical amino acid composition with the substrate-binding domains of other fungi, indicating high conservation. Through comparative analysis, it was also found that four conserved amino acid residues exist in the corresponding positions of BeHMGR: Glu811, Asp1021, His1117, and Ser1123. The presence of these conserved regions and conserved amino acids indicates that the BeHMGR enzyme is likely to be a functional protein.

#### 3.3.3. Phylogenetic Analysis of BeHMGR Proteins

Phylogenetic trees are useful for analyzing the evolutionary relationships between different organisms. Although the structure and function of HMGR proteins are highly conserved during evolution, amino acid counts may vary, allowing for the analysis of evolutionary relationships among different organisms. 

**Figure 2:**
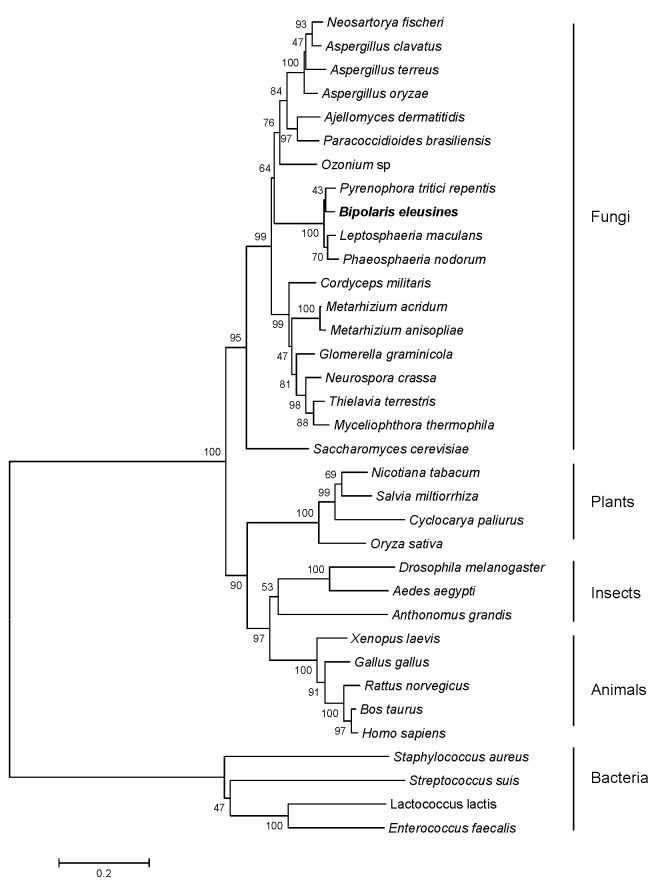
Phylogenetic tree of HMGRs from various species using the Mega 3.1 program by the neighbor-joining method. The bars represent evolutionary distance, and the numbers at each node represent the bootstrap values (with 1000 replicates). The scale bar represents 0.2 nucleotide substitutions per site, indicating the genetic distance between species. Branch lengths are proportional to genetic divergence, allowing for a comparison of the evolutionary genetic distances among the species. The sequences used are listed below with their GenBank accession numbers: *Ozonium* sp., ABU95054; *Leptosphaeria maculans*, CBX91449; *Glomerella graminicola*, EFQ33622; *Metarhizium acridum*, EFY91120; *Metarhizium anisopliae*, EFY99198; *Ajellomyces dermatitidis*, EGE84234; *Cordyceps militaris*, EGX93619; *Neurospora crassa*, XP964546; *Aspergillus terreus*, XP001218142; *Neosartorya fischeri*, XP001265930; *Aspergillus clavatus*, XP001272815; *Phaeosphaeria nodorum*, XP001800116; *Aspergillus oryzae*, XP001823959; *Pyrenophora tritici-repentis*, XP001941036; *Paracoccidioides brasiliensis*, XP002792508; *Thielavia terrestris*, XP003656898; *Myceliophthora thermophila*, XP003658928; *Saccharomyces cerevisiae*, EDV08752; *Anthonomus grandis*, AF162705; *Drosophila melanogaster*, AAA28608; *Aedes aegypti*, XP001659923; *Rattus norvegicus*, AAA40608; *Bos taurus*, DAA25922; *Homo sapiens*, NP000850; *Cyclocarya paliurus*, EU296534; *Nicotiana tabacum*, U60452; *Salvia miltiorrhiza*, FJ747636; *Gallus gallus*, NP989816; *Oryza sativa*, NM001070076; *Xenopus laevis*, AAH74197; *Lactococcus lactis*, NP267726; *Enterococcus faecalis*, ZP05563025; *Staphylococcus aureus*, NP373069; and *Streptococcus suis*, AER19871.

To analyze the molecular evolutionary relationship between BeHMGR and HMGRs from other species, we constructed a molecular phylogenetic tree using the full-length amino acid sequences of HMGRs from bacteria, fungi, animals, and plants. As shown in Figure 2, HMGR proteins share common ancestors, which then diversified into different branches: bacteria, fungi, animals, and plants. Within the fungal branch, yeast evolved earlier than other filamentous fungi. BeHMGR forms a well-defined group with the HMGR of filamentous fungi, and its closest genetic relationship is with *Pyrenophora tritici-repentis*. 

### 3.4. Effect of Methyl Jasmonate on BeHMGR Gene Expression

**Figure 3:**
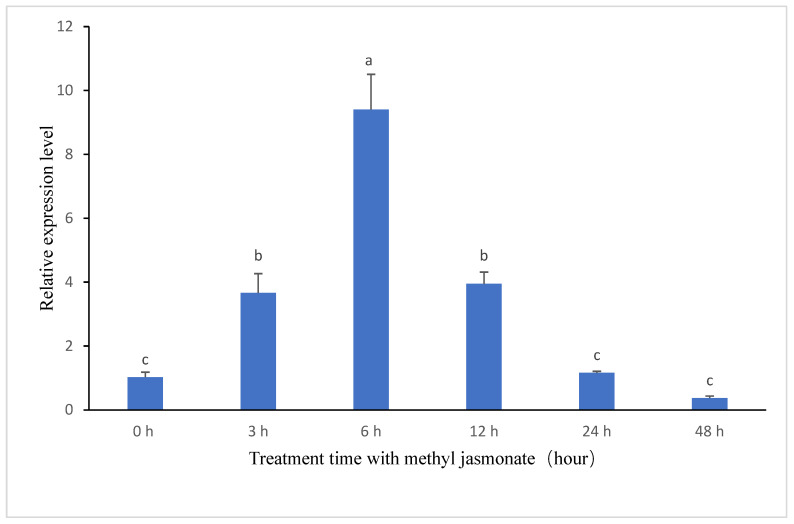
Quantitative real-time PCR analysis of *BeHMGR* gene expression associated with the ophiobolin A biosynthesis pathway. Total RNA was extracted from *Bipolaris eleusnies* mycelia at various time points (0 h, 3 h, 6 h, 12 h, 24 h, and 48 h) following treatment with methyl jasmonate. The actin gene served as the control to demonstrate the normalization of RNA loading in the PCR reaction. Data are expressed using the 2^−∆∆Ct^ method. A standard variance analysis with a completely randomized experimental design, followed by Tukey’s multiple-range tests, was conducted to evaluate statistical differences between treatment times. Data are presented as mean ± SD (*n* = 3). Means with different letters represent Tukey’s honest significant difference at *p* < 0.05.

To investigate the expression of the *BeHMGR* gene in the terpenoid synthesis pathway of *B. eleusines* in response to methyl jasmonate treatment, this study employed quantitative fluorescence PCR to measure the gene’s induced expression. The results demonstrate that *BeHMGR* gene expression was significantly affected by methyl jasmonate at *p *< 0.05. Specifically, the transcript levels of *BeHMGR* increased markedly, showing a significant rise at 3 h post-treatment, peaking at 6 h, and achieving a maximum expression level of 9.2-fold compared to the baseline (0 h) (see Figure 3). Following this peak, the expression levels gradually declined.

## 4. Discussion

Although whole genome sequence data for several *Cochliobolus* (anamorph: *Bipolaris*) species exist in international databases, Condon et al. (2013) reported that the genes encoding polyketide synthase (PKS), which catalyze the synthesis of a class of secondary metabolites closely related to virulence, are remarkably diverse among *Cochliobolus* species but conserved among isolates of the same species [28]. Presently, the complete genome sequence of *B. eleusines* has not been determined, and the genetic biology of this fungus remains largely unknown. To obtain specific information on genes associated with ophiobolin biosynthesis, we conducted transcriptome sequencing of *B. eleusines*. This yielded a total of 26,555,560 high-quality ESTs, and KEGG Pathway analysis identified 60 genes in the terpenoid backbone biosynthesis pathway. A comparison with the database reveals that these pathway gene sequences offer abundant genetic insights for gene cloning and research on the ophiobolin biosynthetic pathway. We identified several key enzyme genes, including *HMGR*, *IDDI*, *FPPS*, and *GGPPS*, in the ophiobolin biosynthetic pathway and first selected the *HMGR* gene for cloning analysis.

The *HMGR* gene plays a critical role in the mevalonate pathway, synthesizing isoprenoids and sterols, including ophibolin A. It has been cloned in numerous species of fungi [23,29,30,31,32,33,34]. In this study, we report the isolation and characterization of the *HMGR* gene from *B. eleusines* using RACE technology. The full-length cDNA of this gene was 3906 base-pairs long, with a 3474 base-pair open reading frame (ORF) encoding 1157 amino acids. Sequence analysis reveals that the deduced BeHMGR shares high similarity with HMGRs from other fungi, including *Pyrenophora tritici-repentis* (93%), *Leptosphaeria maculans* (90%), and *Phaeosphaeria nodorum* (86%).

In eukaryotes, HMGR is an integral membrane glycoprotein located in the endoplasmic reticulum. The N-terminal composition and length of the HMGR protein vary significantly, whereas the functions of certain amino acids at the C-terminal are highly conserved and play a crucial role in the protein’s conformation and catalytic properties [34,35]. These conserved amino acid residues of HMGRs include His924, Glu618, Asp828, and Ser930, which regulate HMGR activity [36,37,38]. Nevertheless, variations in the conserved amino acids among HMGR proteins across various fungal species exist. The C-terminal amino acid sequence of the cassava pathogen *Sphaceloma manihoticola* HMGR contains all conserved amino acids [31], while no serine residue was found at the corresponding position in yeast HMGR [23]. Through comparative analysis, we found that four conserved amino acid residues exist in the corresponding positions of BeHMGR: Glu811, Asp1021, His1117, and Ser1123 (see Supplementary File S2). The number of intervening amino acids between them is entirely consistent with that reported in the literature [31].

Ophiobolin A, like most fungal secondary metabolites, is synthesized in very small amounts under normal physiological conditions. Inducing and regulating the biosynthetic pathway is an effective method for significantly increasing its production. Methyl jasmonate is an important signaling molecule that activates the transcription and translation of specific defense genes, ultimately promoting the synthesis of the secondary metabolite ophiobolin. Numerous studies have reported that the expression of terpenoid synthesis genes can be induced by methyl jasmonate [39,40,41]. This study finds that the expression levels of *BeHMGR* genes were significantly increased after treatment with methyl jasmonate, indicating that they are induced by methyl jasmonate and can be effectively regulated, at least at the transcriptional level.

## 5. Conclusions

The transcriptome sequence of *B. eleusines* was determined, and the first rate-limiting enzyme gene in the ophiobolin A synthesis pathway of *B. eleusines*, *BeHMGR*, was isolated and cloned. The full-length cDNA sequence of the *BeHMGR* gene is 3906 bp, contains an open reading frame of 3474 bp, and encodes a protein with 1157 amino acid residues. Fluorescence quantitative PCR analysis reveals that the expression of the *BeHMGR* gene was induced after methyl jasmonate treatment.

## Figures and Tables

**Table 1:** All primers used in this study.

Primer Name	Primer Sequences (5′-3′)
*BeHMGR*-ECT-F	CGTACCCCCGGCCCAGATGA
*BeHMGR*-ECT-R	CGCGCGAAGTTGAAGCGACG
*BeHMGR*3-1	TCTACCTCTCGCTTCGCCAGGCTACAA
*BeHMGR*3-2	TACCGACAAGAAGTCTGCCGCCATCAA
*BeHMGR*5-GSP1	TGGCGTTGCTGCTCTT
*BeHMGR*5-GSP2	AATCTCTGGCTGGGGTCTTGGC
*BeHMGR*5-GSP3	TACGGGGCGGGGTAGGCATGTG
*BeHMGR*-FL-F	ACAATGCTAGGATCACTCGCCA
*BeHMGR*-FL-R	ACTTTCTCTATCGCTTGGGCAC
*Actin*177F	CATCAACCCCAAGTCCAACC
*Actin*177R	CCCTCGTAGATGGGGACAAC
*HMGR*158F	TGTCCCCGGAACCCCTCGCA
*HMGR*158R	GGCGTTGCTGCTCTTCCGTTG

**Table 2:** Sequencing yield statistics.

Total Reads	Total Nucleotides * (nt)	Q20 Percent	N Percent	GC Percent
26,555,560	2,390,000,400	92.91%	0.00%	50.77%

* Total nucleotides = Total Reads 1 × Reads 1 size + Total Reads 2 × Reads 2 size.

**Table 3:** Consistency sequence (Unigene) statistics obtained by Blast annotation.

Nucleotide Length	100–500 nt	500–1000 nt	1000–1500 nt	1500–2000 nt	≥2000 nt
Number	20,075	7460	3021	1078	466
Percent (%)	62.54	23.24	9.41	3.36	1.45

## Data Availability

Data are contained within the article.

## Supplementary Materials

The following supporting information can be downloaded at: https://www.mdpi.com/article/10.3390/jof10070445/s1, Supplementary File S1: *BeHMGR* gene full-length cDNA sequence and its deduced amino acid sequence; Supplementary File S2: Multiple alignment of the deduced amino acid sequences of BeHMGR and other fungal HMGR proteins.
