# Supplementary material for: Molecular Characterization and Expression Analysis of a Gene Encoding 3-Hydroxy-3-Methylglutaryl-CoA Reductase (HMGR) from Bipolaris eleusines, an Ophiobolin A-Producing Fungus
Source: J Fungi (Basel). 2024 Jun 26;10(7):445. doi: 10.3390/jof10070445 (PMC11277564; doi:10.3390/jof10070445)
Supplement: Supplementary file 1 [file jof-10-00445-s001.zip › supplementary caption.pdf]

Supplementary File S1. *BeHMGR* gene full-length cDNA sequence and its deduced amino acid sequence. The start codon and stop codon are shown in red bold font.

Supplementary File S2. Multiple alignments of the deduced amino acid sequences of BeHMGR and other fungal HMGR proteins. These HMGRs were downloaded from GenBank, and the amino acid sequences were obtained. They include *Ozonium* sp. (accession no. ABU95054), *Leptosphaeria maculans* (accession no. CBX91449), *Glomerella graminicola* (accession no. EFQ33622), *Ajellomyces dermatitidis* (accession no. EGE84234), *Neurospora crassa* (accession no. XP964546), *Aspergillus terreus* (accession no. XP001218142), *Phaeosphaeria nodorum* (accession no. XP001800116), *Pyrenophora tritici-repentis* (accession no. XP001941036), *Saccharomyces cerevisiae* (accession no. EDV08752), *Mycosphaerella_graminicola* (accession no. EGP90820), respectively. Identical amino acid sites are represented by white fonts on black backgrounds, conserved amino acid sites are represented by black fonts on red backgrounds, and similar amino acid sites are represented by black fonts on blue backgrounds. Non-similar amino acids are represented by black fonts on white backgrounds. The two conserved regions that bind NADPH and the two conserved regions that bind HMG-CoA are underlined.
