# Supplementary material for: Molecular Characterization and Expression Analysis of a Gene Encoding 3-Hydroxy-3-Methylglutaryl-CoA Reductase (HMGR) from Bipolaris eleusines, an Ophiobolin A-Producing Fungus
Source: J Fungi (Basel). 2024 Jun 26;10(7):445. doi: 10.3390/jof10070445 (PMC11277564; doi:10.3390/jof10070445)
Supplement: Supplementary file 1 [file jof-10-00445-s001.zip › Supplementary File S1.pdf]

```
1    ATGAGAAAT
10   TGGGGTGTGCCAAGGGAGGGACGGATTGTTGCGAGGGAGCCGAGT
55   TGAGGGTGCTGGACGAGGGGAAGACGAGGGTGACAAGGGCGAGTT
100  CCTGGGCGTTGTCGGCAGCACTGCCTCTGGCGTCCGAGGCCTCCC
145  ACTGCCATGCTGTTTCTTCTCCAACCTCGGTGGTAGACGCCCACA
190  ATGCTAGGATCACTCGCCAGCCGCTGGCAGGCGACGGGCGACCAG
     M  L  G  S  L  A  S  R  W  Q  A  T  G  D  Q
235  CAAAAGACTGCCCCAACCTGGTTCGACCGTCACCTGTTCCCCGTC
     Q  K  T  A  P  T  W  F  D  R  H  L  F  P  V
280  CTGCTCTCGGTAGCCAAGAAGGCATGCACGCATCCCATCCACACC
     L  L  S  V  A  K  K  A  C  T  H  P  I  H  T
325  ATTGTCACCATTGCCGTCCTCGCCAGCTACTCGTACCTCGGCGTC
     I  V  T  I  A  V  L  A  S  Y  S  Y  L  G  V
370  TTGGACAAGGGACTCTTGGAGAGCGGCATCGAGGATGTCTCCAAC
     L  D  K  G  L  L  E  S  G  I  E  D  V  S  N
415  AATGTCGACTTCCAGTCCCTTTTGGCGGGCAGTAAGACACTGAGG
     N  V  D  F  Q  S  L  L  A  G  S  K  T  L  R
460  GTTGGAGAAGAAACAGCATGGCAGTGGGAGGCCTCGGACGCCAGA
     V  G  E  E  T  A  W  Q  W  E  A  S  D  A  R
505  GGCAGTGCTGCCGACAACGCCCAGGAACTCGCCCTTGTCACCCTC
     G  S  A  A  D  N  A  Q  E  L  A  L  V  T  L
550  GTCTTCCCCTTGTCCAGCACCCTCAACTCGGCTCCCTCGCAACAA
     V  F  P  L  S  S  T  L  N  S  A  P  S  Q  Q
595  TCCGTCCCTCAGAATGTGTCCGCACAGCTCCTTCCCAGCTCCTAC
     S  V  P  Q  N  V  S  A  Q  L  L  P  S  S  Y
640  AGCCCCTTCTCAACTCTTTCTCACGATACCTCGCTCGCATACGCT
     S  P  F  S  T  L  S  H  D  T  S  L  A  Y  A
685  ATGCCATACGATGAGGCTGCCAAATTCCTCGAGGCCATGCAAGAG
     M  P  Y  D  E  A  A  K  F  L  E  A  M  Q  E
730  ATCCCCACACCAGAGGATATCACCCAAGCCCAGAGTCCTGCGCAT
     I  P  T  P  E  D  I  T  Q  A  Q  S  P  A  H
775  GAAGGTTCCCGCGAGCAGAAGAAGTGGATGATGCGTGCCTCCAAG
     E  G  S  R  E  Q  K  K  W  M  M  R  A  S  K
820  CACGGCAATCCCCCTTCAGGAATGCGAAACTGGATCGTTGACTCT
     H  G  N  P  P  S  G  M  R  N  W  I  V  D  S
865  TGGACCTCATTCCTTGACCTACTCAAGAATGCCGACACTGGTGAC
     W  T  S  F  L  D  L  L  K  N  A  D  T  G  D
910  ATTGTCATTATGGCCATGGGATACCTTGCCATGCACCTGACGTTC
     I  V  I  M  A  M  G  Y  L  A  M  H  L  T  F
955  GTCTCCCTCTTCCTCGCCATGAGGCGACTGGGTTCCAACTTTTGG
     V  S  L  F  L  A  M  R  R  L  G  S  N  F  W
1000 CTGGCTACTGCTGTTCTATTGCAATCCGCCTTTGCTTTCCTCTTT
     L  A  T  A  V  L  L  Q  S  A  F  A  F  L  F
```

1045 GCCCTCGCTGTGACGACTTACTTCGGTGTCTCTATCAACCTGATC
A L A V T T Y F G V S I N L I
1090 CTTCTCTCCGAAGGACTTCCTTTCCTAGTTGTCATCATCGGATTT
L L S E G L P F L V V I I G F
1135 GAAAAGCCCATTGTTCTCACAAAGGCCGTCTTGTCTGCCTCCTTG
E K P I V L T K A V L S A S L
1180 GACGGCCGACGAGCAGCCGAGGAGAAGCGAGGCGAGCCTGTGACG
D G R R A A E E K R G E P V T
1225 ATTCAAACCGCCGTGCAGACGGCTATCAAGAAGACTGGCTTCGAG
I Q T A V Q T A I K K T G F E
1270 GTTGTTCGTGACTACTTCTTTGAGATTCTGGTTCTCATCGCAGGC
V V R D Y F F E I L V L I A G
1315 GCCTTGTCTGGAATCCAAGGTGGTCTACGCCAGTTCTGTTTCCTA
A L S G I Q G G L R Q F C F L
1360 GGTGCCTGGATTCTCTTCTTCGATGCCCTCATGCTTCTGACCTTC
G A W I L F F D A L M L L T F
1405 TACACGTCCATTCTCACCGTCAAACTTGAGATCAACCGAATTAAG
Y T S I L T V K L E I N R I K
1450 CGTCATGTTGCTCTTCGCCGCGCTCTCGAAGACGATGGCATTGAC
R H V A L R R A L E D D G I D
1495 GGCAAGGTCGCCGAGAGCGTTGCTCGCAGCAACGACTGGCCTAGT
G K V A E S V A R S N D W P S
1540 GCTCGTGATGTGCAGGTCAGCAGCAACAGCACGACTGTCTTTGGA
A R D V Q V S S N S T T V F G
1585 AAGAAGATCACCGTCCCCAAGTTTAAGATTTTTATGGTCGCCGGA
K K I T V P K F K I F M V A G
1630 TTTTTCCTTGTCAACATCCTCAATGTTACCACGCTCAAGTTTGGC
F F L V N I L N V T T L K F G
1675 TTTGCACCTTGCAAGTCCTATTTTGTCTCTGTTGTTGGCTCTACC
F A P C K S Y F V S V V G S T
1720 CCTCCCCTGGATCCTTTCAAGGTTGCTGGAAGTGGCCTGGATCAC
P P L D P F K V A G S G L D H
1765 ATCTTTGAGCAGGCAAAGGAAGCTGCAACATCGACCGTTGTTACT
I F E Q A K E A A T S T V V T
1810 ATCCTCATGCCGATCAAGTACGAGCTCGAGTTCCCTTCGATTCAC
I L M P I K Y E L E F P S I H
1855 TACGCCGAGCCTTCTCTTGCCGACTCAGATCATGCCTTTGGCACT
Y A E P S L A D S D H A F G T
1900 AACATTAGCACTCACATTGTCGATGGCGTGCTTAAGAGTCTGGAA
N I S T H I V D G V L K S L E
1945 GATCCATTCCTTAGCAAGTGGATCATACTTGCGCTTGTCATGAGC
D P F L S K W I I L A L V M S
1990 GTGGTCCTTAACGGTTACCTCTTCAATGCTGCTCGATGGACAATC
V V L N G Y L F N A A R W T I

2035 AAAGAGCCGCACAAGCCACTGGAACCTCCTTCTCCCTCTGAAGTC

K E P H K P L E P P S P S E V

2080 CTGGACGGAGCCCCCACTGTCCCCGGAACCCCTCGCATTCCATCC

L D G A P T V P G T P R I P S

2125 ATGCACATGCCTACCCCGCCCCGTACCCCCGGCCCAGATGAACAA

M H M P T P P R T P G P D E Q

2170 GTCAAGTGTCTCCAGCCACTTACTCAAGTCCAGCCAAGACCCCAG

V K C L Q P L T Q V Q P R P Q

2215 CCAGAGATTCCAGCAGGCCCAACGGAAGAGCAGCAACGCCAGCCT

P E I P A G P T E E Q Q R Q P

2260 AACCGTCCCTACGAGACTCTTGAGCAAATGATCAAGGACAAACAG

N R P Y E T L E Q M I K D K Q

2305 GCCCCTAAGATGACCGATGAGGAACTGATTGAAATGTCGCTAAGG

A P K M T D E E L I E M S L R

2350 GGTAAGATCCCAGGTTACGCACTAGAGAAGACTCTTGGTGATAAG

G K I P G Y A L E K T L G D K

2395 ACTCGCGCTGTCAAGATTCGCCGCGGCCTCGTTTCCCGAACACAC

T R A V K I R R G L V S R T H

2440 GCTACACGAGAAACATCAACTCTGCTGGAACGCTCACTTCTGCCA

A T R E T S T L L E R S L L P

2485 TACAAGGACTACAACTACGATCTTGTCCATGGCGCATGCTGTGAA

Y K D Y N Y D L V H G A C C E

2530 AACGTTGTTGGTTATCTCCCTCTTCCCCTTGGTGTCGCTGGCCCA

N V V G Y L P L P L G V A G P

2575 ATGCTCATTGACGGCCAAAATTACTTCCTCCCCATGGCAACTACT

M L I D G Q N Y F L P M A T T

2620 GAAGGTGTCCTCGTCGCTTCAACTTCGCGCGGTGCAAAGGCTATC

E G V L V A S T S R G A K A I

2665 AACGCTGGTGGCGGTGCCGTTACTGTTATCACTGGCGATGGCATG

N A G G G A V T V I T G D G M

2710 ACTCGTGGACCCTGTATTGGATTCGACAGCCTCGCACGCGCAGGT

T R G P C I G F D S L A R A G

2755 GCTGCCAAGATCTGGCTTGACTCGGAAGAAGGCCAAAGGACCATG

A A K I W L D S E E G Q R T M

2800 AAGGATGCTTTCAACTCTACCTCTCGCTTCGCCAGGCTACAATCG

K D A F N S T S R F A R L Q S

2845 ATGAAATCTGCCATTGCCGGAACCAACATCTACGTTCGCTTCAGG

M K S A I A G T N I Y V R F R

2890 GCCACGACTGGCGATGCCATGGGCATGAACATGATTTCCAAGGGT

A T T G D A M G M N M I S K G

2935 GTCGAGCATGCTCTGAACGTCATGGCCAATGACTGCGGCTTCGAA

V E H A L N V M A N D C G F E

2980 GACATGCGTGTGGTCGCTGTTTCTGGTAATTACTGTACCGACAAG

D M R V V A V S G N Y C T D K

3025 AAGTCTGCCGCCATCAACTGGATCGACGGCCGTGGCAAGGGTGTT
K S A A I N W I D G R G K G V
3070 GTTGCCGAAGCCATGATTCCCGGCTCCGTTGTCCGATCAGTTCTC
V A E A M I P G S V V R S V L
3115 AAGTGTGAAGTCGACGACCTTGTCCAGATGAACATTTCCAAGAAC
K C E V D D L V Q M N I S K N
3160 TTCATCGGCTCAGCCATGGCGGGTGCTATGGGTGGATTCAACGCG
F I G S A M A G A M G G F N A
3205 CACGCTGCGAATATTGTTGCTGCTGTCTTCTTAGCTACCGGTCAA
H A A N I V A A V F L A T G Q
3250 GATCCTGCTCGGGTTGTTGAAAGTGCCAACTGCATTACCATCATG
D P A R V V E S A N C I T I M
3295 AACAATGTCAACGGCAACCTCCAGATTTCGGTTTCTATGCCTTCG
N N V N G N L Q I S V S M P S
3340 ATAGAAGTCGGCACCATTGGTGGCGGAACCATTCTTGAGCCCCAG
I E V G T I G G G T I L E P Q
3385 TCTGCCATGCTCGACCTGCTCGGCGTACGGGGTGCCCACCCAACT
S A M L D L L G V R G A H P T
3430 TCTCCCGGCGACAATGCTCGCCAGCTCGCTCGTGTCATTGCCGCT
S P G D N A R Q L A R V I A A
3475 GGTGTGCTTGCGGGCGAACTGTCGCTTAACAGTGCGCTGTGCGCT
G V L A G E L S L N S A L C A
3520 GGCCACCTGGTCAAGGCACACATGGCACACAACAGGAGTAACGTT
G H L V K A H M A H N R S N V
3565 CCCTCCCGAGCGCCCACACCCGGCACCATGACGCCCGTTGCGTCA
P S R A P T P G T M T P V A S
3610 GGCACTGGACTGAGCCTGATGAATGCAGCTGCAGGAATTGTGCCC
G T G L S L M N A A A G I V P
3655 AAGCGA**TAG**AGAAAGTATAACGTGTAAATGGAGAGGTTTTTATCT
K R *
3700 TTGTACATGTGCATTGTATGATGGGGAGAAACAGCGTCTAGACTT
3745 TTTCCATGCTGAGCTTCCTAACCAAGAAATTCTTCTCCTTTTTTC
3790 TTCTTGGTTTGCACTCACACTTAATTTCCTTTTGGCAATATTCT
3835 TTTCCGCCGGGGCACAAGTTAGCATGCAATACAAACGTTTCTTTT
3880 GTTCAGTAAAAAAAAAAAAAAAAAAAA
