# Supplementary figures and images for: Molecular Characterization and Expression Analysis of a Gene Encoding 3-Hydroxy-3-Methylglutaryl-CoA Reductase (HMGR) from Bipolaris eleusines, an Ophiobolin A-Producing Fungus
Source: J Fungi (Basel). 2024 Jun 26;10(7):445. doi: 10.3390/jof10070445 (PMC11277564; doi:10.3390/jof10070445)

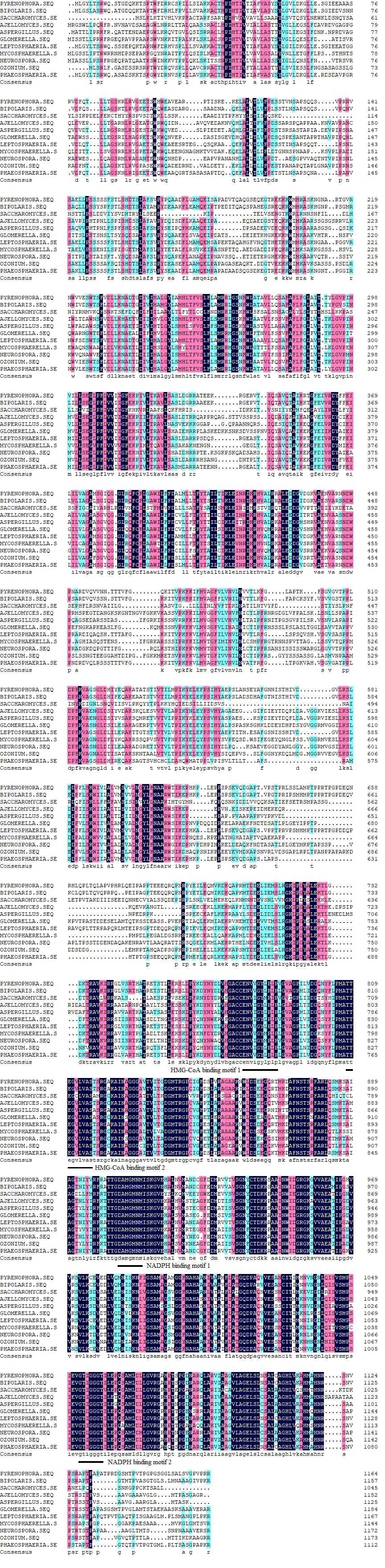

Supplement: Supplementary file 1 [file jof-10-00445-s001.zip › Supplementary File S2.png]
